# Formation of Cl-Doped ZnO Thin Films by a Cathodic Electrodeposition for Use as a Window Layer in CIGS Solar Cells

**DOI:** 10.3390/ma11060953

**Published:** 2018-06-05

**Authors:** Jianping Ao, Rui Fu, Ming-Jer Jeng, Jinlian Bi, Liyong Yao, Shoushuai Gao, Guozhong Sun, Qing He, Zhiqiang Zhou, Yun Sun, Liann-Be Chang

**Affiliations:** 1 Institute of Photoelectronic Thin Film Devices and Technology and Tianjin Key Laboratory of Thin film Devices and Technology, Nankai University, Tianjin 300071, China; aojp@nankai.edu.cn (J.A.); furui4321@163.com (R.F.); bijinlian815@126.com (J.B.); yaoliyong@yeah.net (L.Y.); gaoshoushuai@yeah.net (S.G.); taigic@nankai.edu.cn (G.S.); Heqing27@nankai.edu.cn (Q.H.); zhqzhou@126.com (Z.Z.); suny@nankai.edu.cn (Y.S.); 2 Department of Electronic Engineering, Chang Gung University, Kweishan, Taoyuan 333, Taiwan; liann@mail.cgu.edu.tw; 3 Department of Otolaryngology-Head and Neck Surgery, Chang Gung Memorial Hospital, Kweishan, Taoyuan 333, Taiwan

**Keywords:** Zinc oxide, RF sputtering, electrodeposition, Cl-doped zinc oxide (ZnO:Cl), cadmium sulfide, CIGS solar cells, transmittance

## Abstract

Zinc oxide films that are prepared by radio frequency (RF) sputtering are widely used as window layers in copper indium gallium diselenide (CIGS) solar cells. To reduce their production cost, the electrodeposition method for preparing Cl-doped zinc oxide (ZnO:Cl), rather than sputtering, was studied. The electrodeposition parameters of injected current density and the pH of the electrolyte solution were studied. A moderate current density was used to yield high quality zinc oxides. The pH of the electrolyte greatly affected the formation of ZnO films. The pH value of the electrolyte that ensured that zinc oxides of high quality are obtained was close to seven. Electrodeposited ZnO:Cl films had higher transmittance than ZnO:Al films in the near-infrared region and so they can be used to improve the performance of solar cells. Our experiments revealed that the CIGS solar cells with electrodeposited ZnO:Cl films as a window layer were slightly more efficient than those with sputtered ZnO:Al films.

## 1. Introduction

Zinc oxides that are prepared by radio frequency (RF) sputtering are widely used as a window layer in copper indium gallium diselenide (CIGS) solar cells [1,2,3]. A good window layer must have high electrical conductivity and a low absorption coefficient at large wavelengths [4,5], typically from infrared (IR) to ultraviolet (UV). To realize these properties, zinc oxides must be doped with another element [6]. This doping should be performed without any detrimental effect on the quality of the oxides. Cationic doping by the replacement of zinc with aluminum, indium, or silver has been commonly used to increase electrical conductivity [7,8,9], but anionic doping by the replacement of oxygen with fluorine, chlorine, or phosphor ions has not been widely used [10,11,12,13]. Chlorine may be a good dopant element for improving the physical properties of zinc oxide owing to its abundance, low cost, and valence difference of one electron with O [5]. Cl that is doped into ZnO can donate one electron to the free carriers and easily generate a conductive n-type material [14]. To reduce the cost of production, the electrodeposition method for preparing Cl-doped zinc oxides (ZnO:Cl), rather than sputtering, is proposed.

The key parameters of electrodeposition are injected current density, electrolyte concentration, pH value of the electrolyte solution, deposition time, and deposition temperature, among others. Our recent works [15] have shown that a high injected current density results in the fast deposition, the production of a Zn metal, and large surface roughness. A low current density results in a low deposition rate, a film of poor quality, and uniformity. A moderate current density can yield high-quality zinc oxides. ZnO:Cl films of high quality cannot be obtained using a low or room electrodeposition temperature. A high electrodeposition temperature (>70 °C) results in an excessive deposition rate and poor film quality. Therefore, an electrodeposition temperature of ~70 °C is favored. The concentration of the electrolyte solution is another important factor that influences the electrodeposition. When the Cl-doped ZnO film is electrodeposited, the concentration of zinc chloride (ZnCl_2_) in solution should be as low as possible. A high concentration of ZnCl_2_ can lead to the formation of Zn in the film. A low concentration of ZnCl_2_ is commonly used. Potassium chloride (KCl) is the main source of Cl in the electrodeposition of a ZnO:Cl film. KCl can increase the conductivity of the solution, facilitating the electrodeposition reaction. ZnO:Cl films with good conductivity and high transmittance can be obtained at a KCl concentration of 0.2 M. However, the effect of pH of the electrolytes on the quality of ZnO:Cl films has not yet been studied. Therefore, the performance of CIGS solar cells with a window layer of Cl-doped ZnO films that are electrodeposited using the electrolyte with various pH values is studied herein.

## 2. Experimental Section

A 1 μm-thick molybdenum (Mo) layer was deposited on soda-lime glass by direct current (DC) magnetron sputtering. A CIGS absorber layer (~2 μm) was prepared by three-step evaporation. A CdS buffer layer with a thickness of 50–60 nm was prepared by chemical bath deposition (CBD). Cl-doped ZnO was grown on the stack-layered substrate of glass/Mo/CIGS/CdS in a conventional three-electrode system using a potentiostat. The working, counter, and reference electrodes were glass/Mo/CIGS/CdS, a Zn foil electrode, and a saturated calomel electrode (SCE), respectively. The electrolyte was a mixed solution of 5 mMZnCl_2_ and 0.2 M KCl. The pH value of the electrolyte solution was adjusted by adding low concentrations of hydrogen chloride (HCl) and ammonia. Oxygen gas was continuously supplied to the electrolyte and the electrolyte temperature was kept at 70 °C during electrodeposition. A moderate current density of 0.2 mA/cm^2^ yielded zinc oxides of good quality at a deposition rate of ~7 nm/min. The chlorine concentration of 1–2% in ZnO:Cl films was determined by an energy dispersive spectroscopy (EDS) measurement. A Ni/Al metal grid was deposited on glass/Mo/CIGS/CdS/ZnO:Cl by electron-beam evaporation. CIGS solar cells that contained a window layer of ZnO:Cl film were fabricated. The active area of each of these CIGS solar cells was 0.358 cm^2^.

The structural properties of the films were analyzed using a Philips X’pert PROX-ray diffractometer (Amsterdam, Netherlands) with Cu Kα as the radiation source. Their surface morphology was observed using a scanning electron microscope (SEM, JEOL JSM-6700, Tokyo, Japan) with energy dispersive spectroscopy (EDS). Multiple UV-vis transmittance spectra were recorded using a Shimadzu UV-1800 (Kyoto, Japan) spectrophotometer. The current-voltage (J-V) characteristics of the CIGS solar cells were obtained using a solar simulator under the standard AM1.5 spectrum with an illumination intensity of 1000 W/m^2^ at room temperature. The light intensity of the solar simulator was calibrated using a standard monocrystalline Si reference solar cell.

## 3. Results

**Figure 1:**
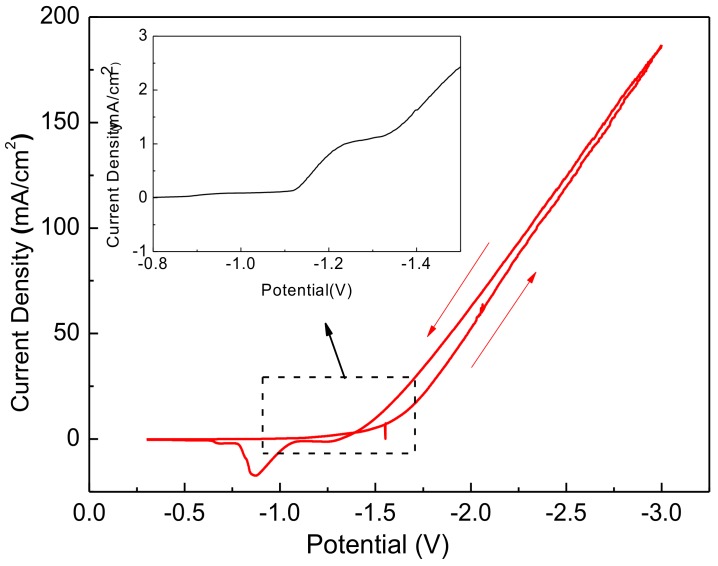
The CV curves of Zn^2+^ions in aqueous solutions with a pH value of 6.5.

Figure 1 plots the cyclic voltammetric (CV) curves of Zn^2+^ ions in aqueous solutions with a pH value of 6.5. The electrolytes mentioned above were used to electrodeposit Cl-doped ZnO films on the stack-layered substrate of the glass/Mo/CIGS/CdS structure. The applied voltage was swept from −0.2 to −3 V and then swept back to −0.2 V. The inset in Figure 1 is a magnification of the curve around −1.2 V for the forward scan. The scanning rate was maintained at 0.02 V/s as the CV curve measurements were made. For the forward scan, the reduction reaction at −1.1 V reduced oxygen molecules to hydroxide ions (Reaction 1). When the applied voltage was more negative than −1.3 V, the cathodic current of the samples in the electrolyte increased, corresponding to the reduction of Zn^2+^ ions in the solution to Zn (Reaction 2). As the applied voltage was swept backward, the oxidation reaction at −0.8 V oxidized Zn to Zn^2+^ (Reaction 3). In the reaction of ZnO at the cathodic electrode, oxygen molecules in the electrolyte gained two electrons, forming hydroxide ions (Reaction 1). The hydroxide ions reacted with Zn^2+^ ions to form zinc hydroxide (Zn(OH)_2_) (Reaction 4). At 70 °C, Zn(OH)_2_ dehydrated spontaneously to ZnO and water (Reaction 5). Therefore, the overall reaction at the cathodic electrode was formulated as Reaction 6. From the CV measurements, a very small electrodepositing current was detrimental to the oxidation and reduction reaction when the applied voltage at the cathodic electrode is less negative than −1.1 V. In that case, the formed ZnO film was non-uniform. However, a very large electrodepositing current caused the formation of Zn. The formation of Zn in the ZnO films seriously degraded the oxide quality. Therefore, choosing the correct applied voltage for electrodeposition is important for obtaining a high-quality ZnO film.

(1) $$\frac{1}{2}O_{2}+H_{2}O+2e^{-}\to 2{\mathrm{OH}}^{-}$$

(2) $${\mathrm{Zn}}^{2+}+2e^{-}\to \mathrm{Zn}$$

(3) $$\mathrm{Zn}-2e^{-}\to {\mathrm{Zn}}^{2+}$$

(4) $${\mathrm{Zn}}^{2+}+2{\mathrm{OH}}^{-}\to \mathrm{Zn}{\left(\mathrm{OH}\right)}_{2}$$

(5) $$\mathrm{Zn}{\left(\mathrm{OH}\right)}_{2}\to \mathrm{ZnO}+H_{2}O$$

(6) $${\mathrm{Zn}}^{2+}+\frac{1}{2}O_{2}+2e^{-}\to \mathrm{ZnO}$$

The growth of ZnO was affected by the pH value of the electrolytes. When the pH of the electrolytes was too low, the reaction products of Zn(OH)_2_ in reaction 4 were consumed, and the formed ZnO dissolved again. The amount of ZnO did not increase. Simultaneously, CdS film dissolves, negatively affecting device performance. However, an excessive pH value promoted the hydrolysis of ZnCl_2_. The Zn(OH)_2_ in the electrolyte solution suppressed reaction 4, so the amount of the ZnO that was produced was insufficient for reaction 5 owing to the hydrolyzability of ZnCl_2_. In the preparation of the electrolytes, a floc was likely to appear in solution. The pH of the electrolyte solution must be adjusted to ensure that the solution is clear. A low concentration of HCl was dropped into the solution. When the floc in the solution disappeared and the solution became clear, the pH of the solution was approximately 6.4. The solutions were rendered weakly acidic or weakly alkaline by dropping low concentrations of HCl or ammonia, respectively, into the solution.

**Figure 2:**
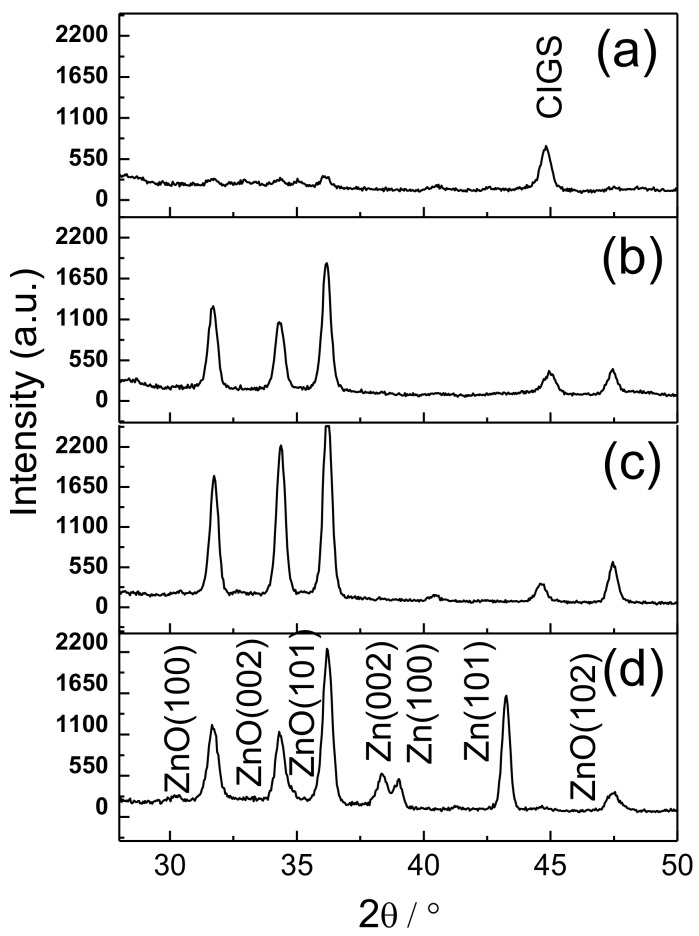
X-ray diffraction (XRD) patterns of Cl-doped ZnO films that were electrodeposited with current densities of (**a**) 0.05, (**b**)0.1, (**c**) 0.2, and (**d**) 0.4 mA/cm^2^.

To find a suitable electrodepositing current for preparing high-quality ZnO films, Figure 2a–d show the X-ray diffraction (XRD) patterns of Cl-doped ZnO films that were electrodeposited with current densities of 0.05, 0.1, 0.2, and 0.4 mA/cm^2^, respectively. The Cl-doped ZnO films yielded three strong peaks that were indexed as (100), (002), and (101), as well as a weak peak that was indexed as (102), indicating a hexagonal wurtzite structure. A slight shift in these peaks toward lower diffraction angles was associated with the Cl-doped ZnO films, indicating that chlorine was incorporated into the crystal structure, expanding the ZnO crystal lattice. At a low current density in Figure 2a, very weak peak intensities were obtained, revealing that Cl-doped ZnO films are very thin and have very poor crystallinity. At a moderate current density, as seen in Figure 2b,c, the peaks were very strong, and no additional peaks that correspond to Zn, Cl, or any other impurity were visible. Cl-doped ZnO films exhibited good crystallinity. At a high current density, as shown in Figure 2d, the peak intensities were strong, but weaker than those in Figure 2c. Three metal Zn peaks, indexed as (100), (002), and (101) were also obtained, revealing that Zn^2+^ ions at the cathodic surface were reduced to Zn and then incorporated into Cl-doped ZnO films. The formation of Zn in Cl-doped ZnO films seriously degraded the oxide quality. Therefore, as indicated above, the current density of 0.2 mA/cm^2^ was suitable for the electrodeposition of Cl-doped ZnO films on the stack-layered substrate of glass/Mo/CIGS/CdS.

**Figure 3:**
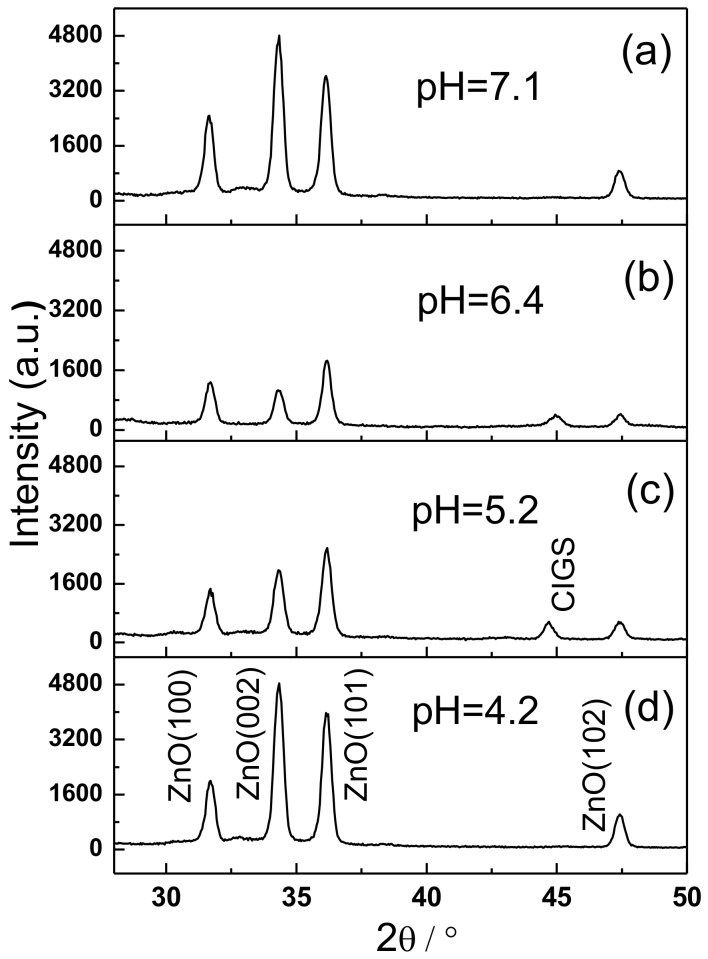
The XRD patterns of Cl-doped ZnO films that were electrodeposited using electrolytes with four pH values of (**a**) 7.1, (**b**) 6.4, (**c**) 5.2, and (**d**) 4.2.

As is well known, ZnO and CdS thin films dissolve in strong acid, detrimentally affecting the performance of CIGS solar cells [16]. Hydrogen bubbles are easily generated in the electrolyte when applying a strong acid owing to the presence of excess hydrogen ions. These bubbles cause the oxide to be of poor quality. Therefore, a weakly acidic electrolyte was used to study the formation of Cl-doped ZnO films. Figure 3a–d show the XRD patterns of Cl-doped ZnO films that were electrodeposited using the electrolytes with pH values of 7.1, 6.4, 5.2, and 4.2, respectively. The thickness of Cl-doped ZnO films was controlled at ~500 nm by adjusting their deposition time, because growth rate varied with pH values. Clearly, the Cl-doped ZnO films with three strong peaks indexed as (100), (002), and (101), and a weak peak indexed as (102), when obtained using electrolytes with various pH values, had a hexagonal wurtzite structure. Interestingly, it was observed that Cl-doped ZnO films, which were prepared by the electrolytes with pH values of 7.1 and 4.2, have a dominant peak of (002), indicating that these ZnO:Cl films grew preferably in [0001] direction, perpendicular to the substrate. However, the Cl-doped ZnO films, which were prepared by the electrolytes with pH values of 6.4 and 5.2, have a dominant (101) peak, and two peaks ((100) and (002)) of moderate intensity. The dominant (101) peak indicates that the ZnO:Cl films may have a random growth direction, independent of the substrates in these samples.

**Figure 4:**
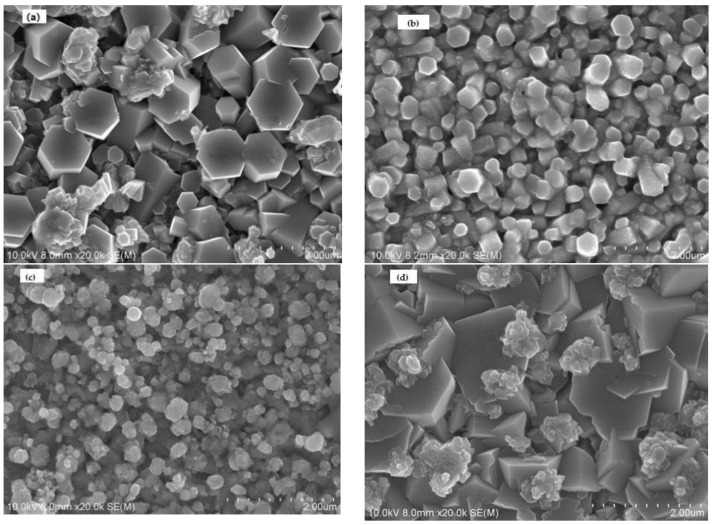
The surface SEM images of Cl-doped ZnO films that were electrodeposited using electrolytes with four pH values of (**a**) 7.1,(**b**) 6.4, (**c**) 5.2, and (**d**) 4.2.

The surface morphologies of the Cl-doped ZnO thin films that were obtained by electrodeposition using solutions with various pH values were studied. Figure 4 shows surface images of Cl-doped ZnO films that were electrodeposited using electrolytes with pH values of 4.2, 5.2, 6.4, and 7.1. As is clearly seen in these figures, the surface morphology had a grained structure and the grains had hexagonal shapes. The films that were deposited at pH = 7.1 exhibited relatively high crystallization, a clear hexagonal columnar structure, obvious grain boundaries, and few clusters. The films that were deposited at pH = 5.2 and pH = 6.4 had smaller grains, a more compact arrangement of grains, and a smooth grain boundary. At a pH value of 4.2, the grain size of the obtained films was larger and the grain boundaries were clear. However, the films contained more clusters, with a disordered arrangement, between the structure and the grains. The growth direction was not uniform. The different growth orientations of the ZnO thin films may have arisen from the various mechanisms of formation under various pH conditions.

**Figure 5:**
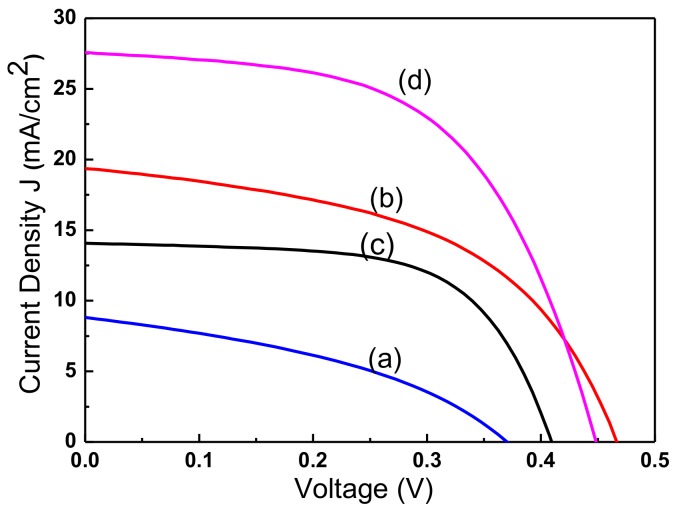
The current-voltage curves of CIGS solar cells with a window layer of Cl-doped ZnO films that were electrodeposited using electrolytes with four pH values of (**a**) 4.2, (**b**) 5.2, (**c**) 6.4, and (**d**) 7.1.

Figure 5 plots the current-voltage curve of CIGS solar cells with a window layer of Cl-doped ZnO films that were electrodeposited using the electrolytes with the four pH values. The cell with Cl-doped ZnO films that was prepared using the electrolytes with a pH value of 7.1 had the highest solar efficiency. In contrast, pH = 4.2 yielded the lowest efficiency. The morphology of the ZnO:Cl films that were obtained at pH = 7.1 had a hexagonal columnar structure with dense grains of moderate size and a clear orientation. The hexagonal columnar structure of the ZnO:Cl film that was obtained at pH = 4.2 is not obvious. The grains were larger and their arrangement was irregular, indicating that the surface morphology of electrodeposited ZnO:Cl films greatly affected the performance of the cell in which they are used. Table 1 presents the detailed performance parameters of CIGS solar cells with a window layer of Cl-doped ZnO films that are electrodeposited using electrolytes with four pH values.

**Figure 6:**
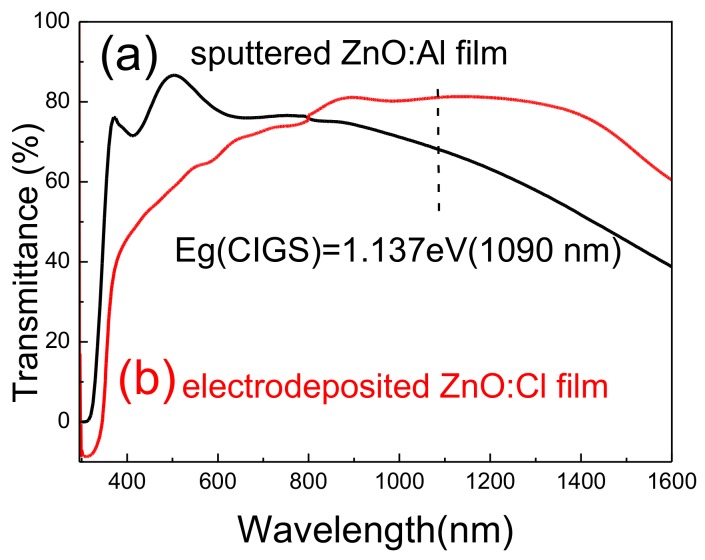
The transmittance of (**a**) sputtered ZnO:Al, and (**b**) electrodeposited ZnO:Cl films.

**Figure 7:**
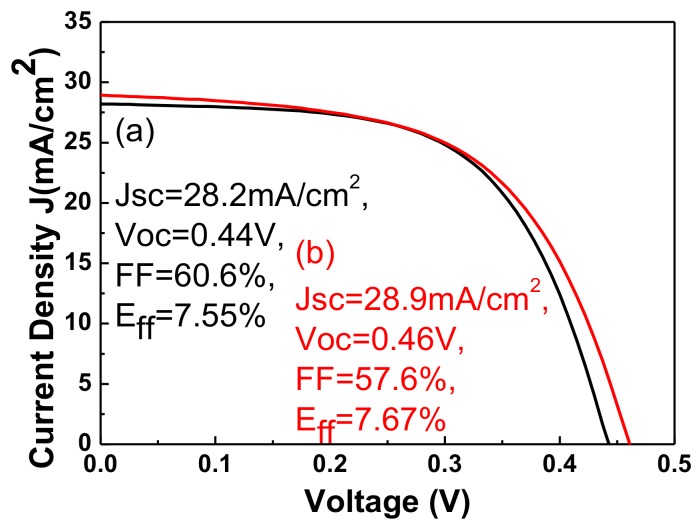
The current-voltage (CV) curves of CIGS solar cells with the window layer of (**a**) sputtered ZnO:Al, and (**b**) electrodeposited ZnO:Cl films.

Figure 6 plots the transmittance of sputtered Al-doped ZnO (ZnO:Al) and electrodeposited Cl-doped ZnO thin films. The thicknesses of Al-doped and Cl-doped ZnO films are 500 and 550 nm, respectively. The transmittance of ZnO:Cl films increased from 50% to 80% as the wavelength increased from 400 to 800 nm, and remained at ~80% for wavelengths ranging from 800 to 1400 nm. The transmittance began to decline when the wavelength reached 1400 nm. A sputtered Al-doped zinc oxide was prepared for comparison. The sputtered ZnO:Al films had a higher transparency than the electrodeposited zinc oxide for wavelengths of 400–800 nm. However, the transmittance of the sputtered ZnO:Al films was much lower in the near-infrared region (>800 nm). The electrodeposited zinc oxide with Cl-doping had high transmittance in the near-infrared region (>800 nm). Therefore, CIGS solar cells with a window layer of ZnO:Cl films exhibit better light absorption in the near-infrared region than do ZnO:Al films. Generally, the resistivity of electrodeposited ZnO:Cl films was higher than those of the sputtered one. However, the transmittance property of ZnO:Cl films is better than that of sputtered ZnO:Al films. A trade-off between the electrical performance and the transparency of the layer needs to be made [17]. Figure 7 plots the current-voltage curve of CIGS solar cells with a window layer of sputtered ZnO:Al and electrodeposited ZnO:Cl films. The efficiency of CIGS solar cells with electrodeposited ZnO:Cl films is slightly higher than that of those with sputtered ZnO:Al films. The low open-circuit voltage of sputtered zinc oxide might have been caused by sputtering damage, which is the creation of recombination centers. The slightly higher short-circuit current in the electrodeposited ZnO:Cl film may have been a result of high absorption in CIGS films in the near-infrared region.

## 4. Conclusions

The electrodeposition parameters of injected current density and the pH value of the electrolyte solution that are used in preparing Cl-doped ZnO films were studied. The XRD results indicated that Cl-doped ZnO films were very thin and had very poor crystallinity at a low current density of 0.005 mA/cm^2^, and high crystallinity at a moderate current density of 0.2 mA/cm^2^. The formation of Zn in Cl-doped ZnO films at a high current density of 0.4 mA/cm^2^ seriously degraded the quality of the oxide. The Cl-doped ZnO films with three strong peaks indexed as (100), (002), and (101), and a weak peak indexed as (102) meant they had a hexagonal wurtzite structure. The dominant peak of (002) from Cl-doped ZnO films that were prepared using electrolytes with pH values of 7.1 and 4.2 showed that in most of the ZnO films, the [0001] direction was perpendicular to the substrate. In contrast, the XRD patterns of the Cl-doped ZnO films that were prepared using electrolytes with pH values of 6.4 and 5.2 showed the (101) peak as dominant, and two peaks ((100) and (002) peaks) of moderate intensity that could have arisen because of the random direction relative to the substrates in these samples. The sputtered ZnO:Al films had a greater transparency to wavelengths of 400–800 nm than does the electrodeposited ZnO:Cl. However, the transmittance of sputtered ZnO:Al films was much lower in the near-infrared region (>800 nm). Therefore, the electrodeposited ZnO:Cl films had a higher transmittance than the ZnO:Al films in the near-infrared region. The experimental results herein indicated that CIGS solar cells with electrodeposited ZnO:Cl films as the window layer were slightly more efficient than those with sputtered ZnO:Al films.

## Figures and Tables

**Table 1:** Performance parameters of CIGS solar cells with Cl-doped ZnO films that were prepared by various pH values.

pH	Voc (mV)	Jsc (mA/cm^2^)	FF (%)	H (%)
4.2	370	8.18	38.98	2.27
5.2	467	19.35	50.21	4.54
6.4	409	14.07	62.70	3.61
7.1	448	27.56	56.12	6.93
